# Crosstalk between signals initiated from TLR4 and cell surface BAFF results in synergistic induction of proinflammatory mediators in THP-1 cells

**DOI:** 10.1038/srep45826

**Published:** 2017-04-04

**Authors:** Su-Geun Lim, Jae-Kwan Kim, Kyoungho Suk, Won-Ha Lee

**Affiliations:** 1 School of Life Sciences, BK21 Plus KNU Creative BioResearch Group, Kyungpook National University, Daegu 41566, Republic of Korea; 2 Department of Pharmacology, Brain Science & Engineering Institute, BK21 Plus KNU Biomedical Convergence Program, Kyungpook National University School of Medicine, Daegu 41944, Republic of Korea

## Abstract

Cellular response to stimulation is mediated by meshwork of signaling pathways that may share common signaling adaptors. Here, we present data demonstrating that signaling pathways initiated from the membrane-bound form of B-cell activating factor (BAFF) can crosstalk with lipopolysaccharide (LPS)-induced signaling for synergistic expression of proinflammatory mediators in the human macrophage-like cell line THP-1. Co-treatment of the cells with BAFF-specific monoclonal antibody and LPS resulted in enhanced mitogen-activated protein kinase (MAPK)/mitogen- and stress-activated protein kinase (MSK)-mediated phosphorylation of nuclear factor kappa-light-chain-enhancer of activated B cells (NF-κB) p65 subunit (Ser276), which then interacts with CREB binding protein (CBP) for subsequent acetylation. Simultaneously, the phosphorylation of cyclic AMP-response element binding protein (CREB) was enhanced through the combined action of phosphatidylinositol-3-kinase (PI3K)/AKT and MAPK/MSK pathways, and the resulting phospho-CREB interacted with the NF-κB/CBP complex. Transfection of CREB-specific siRNA inhibited the BAFF-mediated enhancing effect indicating that the formation of the CREB/NF-κB/CBP complex is required for the synergistic induction of the proinflammatory genes. These findings indicate that BAFF-mediated reverse signaling can modulate LPS-induced inflammatory activation through regulation of NF-κB and CREB activity and point out the necessity to re-evaluate the role of BAFF in diseases where its expression is high in macrophages.

As a member of tumor necrosis factor (TNF) superfamily (TNFSF), B-cell activating factor of the TNF family (BAFF, TALL-1, THANK, BlyS, TNFSF13b, zTNF-4) is involved in B-cell survival^1^ and the pathogenesis of autoimmune diseases^1^,^2^. Various cell types including myeloid cells (monocytes, macrophages, neutrophils, and dendritic cells), stromal cells within lymphoid organs^3^, and osteoclasts^2^,^4^ express both membrane-bound and soluble forms of BAFF. There are three known receptors of BAFF: transmembrane activator and a calcium-modulating cyclophilin ligand interactor (TACI), B-cell maturation antigen (BCMA), and BAFF receptor (BAFF-R, BR3). These receptors are expressed mainly on B cells, plasma cells and T subsets^4^,^5^. Macrophages express of BAFF has been shown to be upregulated in human diseases such as chronic gastritis^6^, atherosclerosis^7^, and acute hepatitis C virus infection^8^. In a mouse model of systemic lupus erythematosus, macrophage expression of BAFF was shown to be induced by the action of interferons and estrogen^9^.

Recently, BAFF has been shown to mediate reverse signaling when BAFF-expressing cells were stimulated with appropriate counterparts or BAFF-specific antibodies in monocyte/macrophage cell lines, as well as in primary mouse macrophage culture^10^. This type of ligand-mediated signaling is a unique property of the TNFSF, which can be expressed on the cell surface as type II transmembrane proteins (reviewed in ref. 11). Members of the TNFSF that can mediate reverse signaling are TNFSF14 (LIGHT)^12^,^13^, TNFSF5 (CD40L)^14^, TNFSF9 (4-1BBL)^15^, TNFSF11 (TRANCE)^16^, TNFSF8 (CD30L)^17^, TNFSF6 (FasL)^18^,^19^, and TNFSF10 (TRAIL)^20^.

This reverse signaling initiated by members of the TNFSF appears to crosstalk with Toll-like receptor (TLR)-mediated signaling. In the case of 4-1BBL, stimulation of it resulted in enhancement of the lipopolysaccharide (LPS)-induced activation of TNF-α and interleukin (IL)-6 in macrophages and dendritic cells^21^. A recent report indicated that transmembrane protein 126 A (TMEM126A), a novel 4-1BBL binding protein, is required for LPS-induced late-phase Janus kinase (JAK) and interferon regulatory factor-3 (IRF-3) phosphorylation^22^. We explored the possibility of crosstalk between signaling pathways initiated by BAFF and Toll-like receptor (TLR)4, a well-known receptor for LPS, in the human macrophage-like cell line THP-1. Simultaneous stimulation of BAFF and TLR4 resulted in synergistic activation of the cells with respect to the expression of proinflammatory mediators such as cytokines and matrix degrading enzymes. The underlying mechanism responsible for this crosstalk was subsequently investigated.

## Results

### Simultaneous stimulation of BAFF and TLR4 synergistically induce the expression of pro-inflammatory mediators

**Figure 1:**
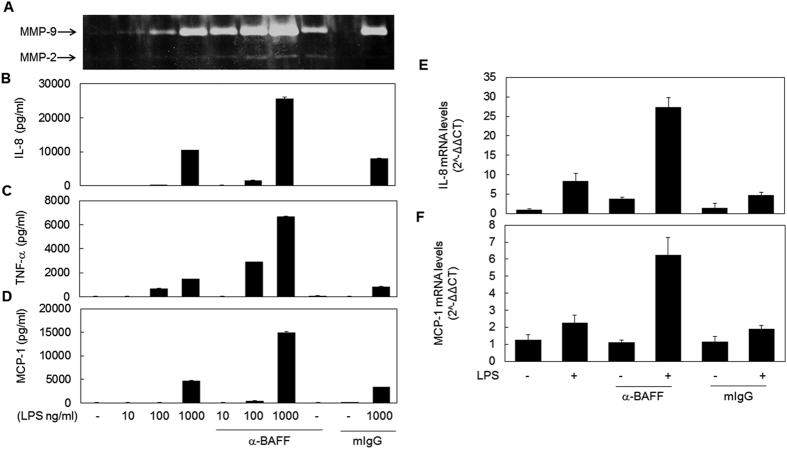
Stimulation of BAFF resulted in the enhancement of LPS-induced expression of pro-inflammatory mediators. (**A–D**) THP-1 cells were pre-treated with 1 μg/ml anti-BAFF mAb or isotype-matching control mouse antibody (mIgG) for 30 min and then stimulated with indicated concentrations of LPS for 9 h (**C**) or 24 h (**A**,**B** and **D**). Levels of MMP-9 secreted in culture supernatant were evaluated by gelatin zymography (**A**). Secretion levels of cytokines were measured using ELISA (**B**–**D**). (**E** and **F**) THP-1 cells were pre-treated as in **A** and then stimulated with 1 μg/ml LPS for 2 h. Expression levels of IL-8 mRNA (**E**) and MCP-1 mRNA (**F**) were measured by quantitative RT- PCR.

In order to test whether TLR4- and BAFF-mediated signaling pathways interact and to determine the effect of this crosstalk in macrophage inflammatory responses, THP-1 cells were pre-treated with anti-BAFF monoclonal antibody (mAb) for 30 min and then stimulated with various doses of LPS. After stimulation, the levels of secreted pro-inflammatory mediators such as matrix metalloproteinase (MMP)-9, TNF-α, IL-8, and macrophage chemoattractant protein (MCP)-1 were evaluated using gelatin zymography or ELISA. As shown in Fig. 1A–D, treatment with a low dose (1 μg/ml) of anti-BAFF mAb alone resulted in the induction of little or no expression of these inflammatory mediators. Pretreatment with anti-BAFF mAb exerted a strong enhancing effect on LPS-induced expression of these pro-inflammatory mediators. This synergistic effect was also observed when the cells were treated with the antibody simultaneously with, and even 30 min after, LPS stimulation (data not shown).

In order to test whether this synergistic induction of proinflammatory mediators occurred at the transcriptional level, mRNA levels of IL-8 and MCP-1 were analyzed using quantitative RT-PCR (Fig. 1E and F). The LPS-induced increase in cytokine mRNA levels was enhanced by pretreatment with BAFF-specific mAb. These results indicate that the synergistic effect of anti-BAFF mAb and LPS co-treatment manifests through transcriptional activation, and that the mechanism of synergism most likely lies in the integration of signals initiated from TLR4 and BAFF.

**Figure 2:**
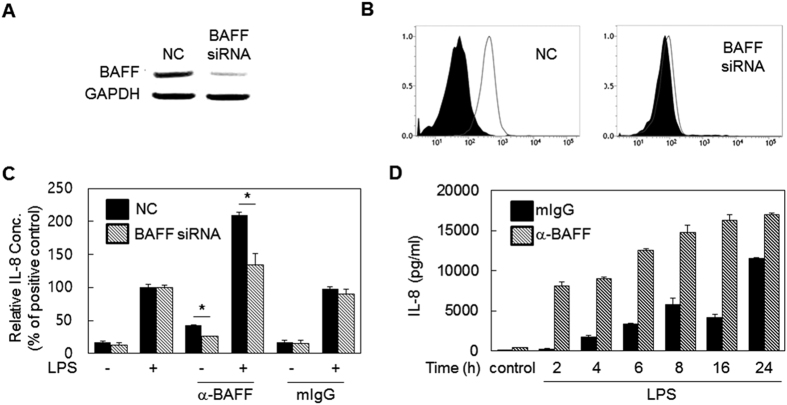
The enhancing effect of mAb is BAFF specific and starts at relatively early time points after LPS stimulation. (**A**) THP-1 cells were transiently transfected with 10 nM scramble siRNA (NC) or BAFF-specific siRNA. After 24 h, RT-PCR analysis was performed to evaluate the expression levels of BAFF mRNA. (**B**) Cells were transfected as in (**A**) and collected at 48 h. BAFF expression levels were then measured by flow cytometry using BAFF specific mAb (empty area) or mIgG (filled area). (**C**) Cells were transfected as in (**A)**. After 48 h, cells were pre-treated with 1 μg/ml mIgG or anti-BAFF mAb for 30 min and then the cells were stimulated with 100 ng/ml LPS for 24 h. Cultured supernatants were then collected to evaluate levels of secreted IL-8. Values represent IL-8 levels relative to those of the positive control (set to 100%). *p < 0.05 (n = 3). (**D**) THP-1 cells were pretreated as in (**C)** and stimulated with 1 μg/ml of LPS. Culture supernatants were collected at the indicated time points and IL-8 levels were measured using ELISA. Control samples were treated with 1 μg/ml anti-BAFF or mIgG for 24 h in the absence of LPS treatment.

To exclude any off-target effects of the antibody on the enhancement of LPS-induced responses, THP-1 cells were transfected with BAFF-specific siRNA. Cells transfected with BAFF-specific siRNA, but not control siRNA, exhibited a marked reduction in BAFF expression which was revealed at both the mRNA (Fig. 2A) and protein levels (Fig. 2B). Then, the transfected cells were stimulated with anti-BAFF mAb and LPS for the measurement of IL-8 expression levels (Fig. 2C). Synergistic induction of IL-8 expression was significantly decreased in cells transfected with BAFF-specific siRNA when compared to those transfected with control siRNA.

THP-1 cells were pre-treated with anti-BAFF mAb, and LPS-induced IL-8 expression levels were measured at various time points. The enhancing effect of the antibody was observed at all time points. Interestingly, the enhancing effect was prominent even as early as 2 h, when LPS-induced expression is relatively low (Fig. 2D).

### LPS-induced activation of ERK and JNK MAPK is enhanced by the stimulation of BAFF

**Figure 3:**
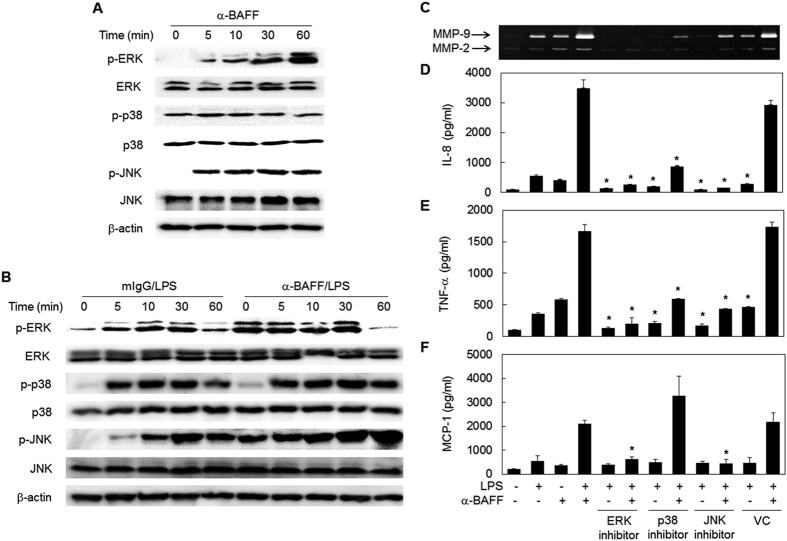
BAFF exerts its enhancing effects through activation of ERK and JNK MAPKs. (**A**) THP-1 cells were stimulated with anti-BAFF (1 μg/ml) mAb for the indicated time periods. The activation status of MAPK was analyzed by Western blot using antibodies specific for normal and phosphorylated form of MAPKs and actin. (**B**) The cells were pre-treated with 1 μg/ml of anti-BAFF mAb or mIgG for 30 min and then stimulated with 100 ng/ml of LPS for the indicated time periods. The Western blot analysis of MAPKs was performed as in **A**. (**C**-**F**) THP-1 cells were pre-treated with 3.75 μM U0126 (ERK inhibitor), 2 μM SB203580 (p38 inhibitor), 10 μM SP600125 (JNK inhibitor) or 0.02% DMSO (VC) for 30 min. Cells were then treated with 1 μg/ml anti-BAFF for 30 min before stimulation with 100 ng/ml of LPS for 24 h. Culture supernatants were collected and levels of secreted MMP-9 (**C**), IL-8 (**D**), TNF-α (**E**) and MCP-1 (**F**) were analyzed by gelatin zymography and ELISA. *p < 0.05 when compared with corresponding positive control samples stimulated in the absence of inhibitors (n = 3).

Mitogen-activated protein kinases (MAPKs) are well-known signaling adaptors involved in the inflammatory activation of macrophages. In order to determine whether MAPKs are involved in the synergistic responses, phosphorylation levels of p38, extracellular signal regulated protein kinase (ERK), and c-Jun N-terminal kinase (JNK) were assessed by Western blot. Anti-BAFF mAb treatment alone induced phosphorylation of ERK and JNK within 5 min while phosphorylation levels of p38 did not change (Fig. 3A). When THP-1 cells were pretreated with antibody, LPS-induced phosphorylation of ERK and JNK, but not those of p38, was enhanced (Fig. 3B). Accordingly, treatment with ERK and JNK-specific inhibitors completely blocked the expression of MMP-9 and cytokines (Fig. 3C–F). The p38-specific inhibitor, however, mildly affected the synergistic expression of IL-8 and TNF-α, probably because it affects TLR4-mediated signaling. In the case of MCP-1 expression, a slight increase was detected in the presence of the p38-specific inhibitor. These results indicate that the synergistic expression of inflammatory mediators is closely associated with enhanced activation of ERK and JNK MAPKs.

### Signal integration culminating in NF-κB activation is responsible for the synergistic induction of pro-inflammatory mediators

**Figure 4:**
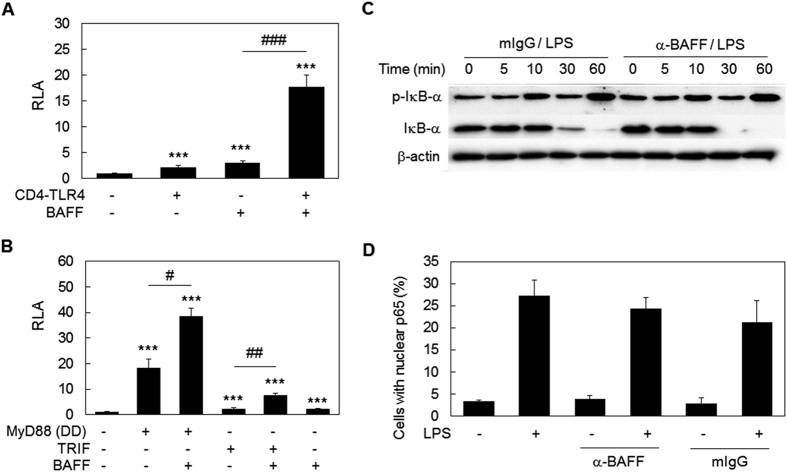
BAFF-mediated signaling synergizes with LPS for NF-κB activation without affecting IκB degradation or NF-κB nuclear translocation. (**A** and **B**) HEK 293 T cells were transfected with an NF-κB luciferase construct, reference reporter construct, and BAFF expression vector in combination with CD4-TLR4, death domain (DD) of MyD88, or wild type TRIF-expressing vectors for 24 h. Relative luciferase activity (RLA) was then measured. ***p < 0.001 when compared with the negative control. ^#^p < 0.05, ^##^ < 0.01 and ^###^ < 0.001 (n = 6). (**C**) THP-1 cells were pre-treated with 1 μg/ml of anti-BAFF mAb or mIgG and then stimulated with 100 ng/ml of LPS for the indicated time periods and total cell lysates were obtained. The Western blot was performed using antibodies specific for phospho-IκB-α, IκB-α or β-actin. (**D**) Cells were pretreated as in C and stimulated with 1 μg/ml of LPS. After 30 min, the location of p65 was analyzed using immunocytochemistry and the proportion of cells with nuclear p65 was determined (n = 3).

Since nuclear factor kappa-light-chain-enhancer of activated B cells (NF- κB) is a key regulator of inflammatory gene expression, the activation status of NF-κB was then analyzed using NF- κB-luciferase assay. A luciferase reporter construct under the regulation of a promoter containing NF-κB binding sites were transfected into HEK 293 T cells along with BAFF expression constructs and/or CD4-TLR4, a constitutively active (CA) form of TLR4^23^. The expression of BAFF after transfection was confirmed by immunofluorescence assay with BAFF specific mAb (data not shown). When both BAFF and CD4-TLR4 expression constructs were transfected together, strong induction of NF-κB activity was detected, and the extent of induction was much higher than the sum of each transfection (Fig. 4A). TLR4-mediated activation of NF-κB mainly involves myeloid differentiation primary response gene 88 (MyD88) and, to a lesser extent, TIR-domain-containing adapter-inducing interferon-β (TRIF)^24^,^25^. The death domain of MyD88, a constitutively active mutant of MyD88^26^, was then used instead of CD4-TLR4. As shown in Fig. 4B, MyD88-mediated NF-κB activation was also the subject of synergism with BAFF. Likewise, TRIF-mediated NF-κB activation, achieved by overexpression of wild type TRIF^27^, was also boosted by BAFF co-transfection (Fig. 4B). These data indicate that BAFF exerts its synergistic effect on NF-κB activation through both MyD88- and TRIF-mediated signaling pathways.

Phosphorylation/degradation of inhibitor of NF-κB (IκB) and the subsequent nuclear translocation of NF-κB is one of the major mechanisms regulating NF-κB activity. In order to determine whether the crosstalk between BAFF- and TLR4-mediated signaling involves IκB in the enhancement of NF-κB activity, IκB phosphorylation and degradation were evaluated. Contrary to expectations, there were no significant differences between cells treated with control antibody or with anti-BAFF mAb, in terms of total protein and phosphorylation levels of IκB (Fig. 4C). In addition, nuclear translocation of NF-κB was not affected by simultaneous stimulation of TLR4 and BAFF (Fig. 4D). These results indicate that the crosstalk between TLR4- and BAFF-mediated signaling results in the enhancement of NF-κB activity without affecting IκB phosphorylation/degradation and the subsequent nuclear translocation of NF-κB.

### Simultaneous stimulation of TLR4 and BAFF causes an increase in Ser276 phosphorylation of NF-κB p65 subunit (RelA), which then interacts with CBP

The activity of NF-κB can be regulated via various other mechanisms, apart from its interaction with IκB and nuclear translocation. These additional regulatory mechanisms include post-translational modification of NF-κB, such as phosphorylation, acetylation, and methylation^28^. Phosphorylation levels at Ser276 of NF-κB p65 subunit (RelA) affect the interaction with co-activator CREB binding protein (CBP), while phosphorylation of Ser536 is associated with its transcription activator function^29^,^30^.

**Figure 5:**
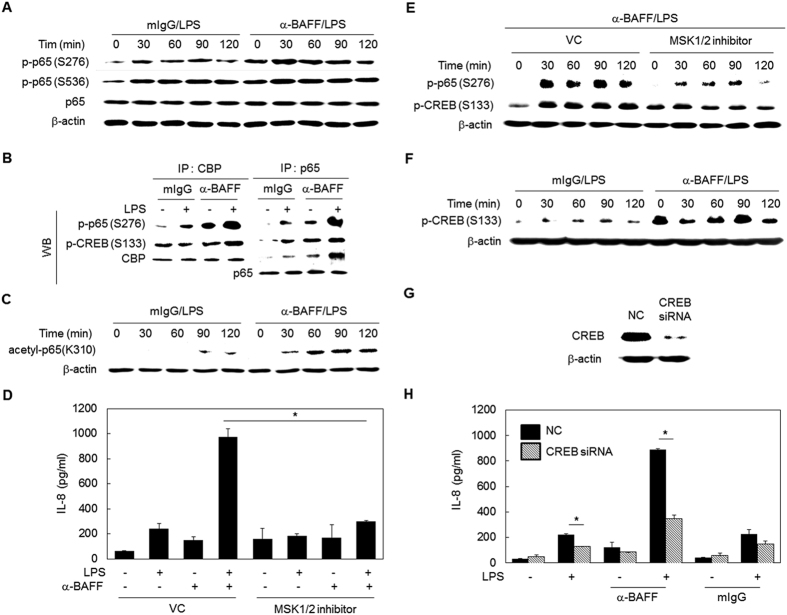
Crosstalk between BAFF- and TLR4-mediated signaling enhanced phosphorylation of NF-κB and CREB for their subsequent interaction with CBP. (**A**) THP-1 cells were pre-treated with 1 μg/ml of mIgG or anti-BAFF mAb for 30 min and then stimulated with LPS (100 ng/ml) for the indicated times. The Western blot analysis was performed using antibodies specific to phospho-p65 (Ser276 or Ser536), p65, and β-actin. (**B**) Cells were treated as in A. After 1 h of LPS stimulation, an immunoprecipitation assay was performed with CBP- or p65-specific antibodies. Western blot analysis was then performed for phospho-p65 (Ser276), phospho-CREB(Ser133), CBP, and p65. (**C**) Cells were treated as in A and the levels of acetyl-p65(Lys310) and β-actin were determined by Western blot. (**D**) THP-1 cells were pre-treated with 5 μM of SB747651A (MSK1/2 inhibitor) or 1% PBS for 1 h. The cells were treated with 1 μg/ml of anti-BAFF for 30 min and then with 100 ng/ml of LPS for 24 h. The levels of secreted IL-8 in culture supernatants were determined by ELISA. *p < 0.05 (n = 3). (**E**) Cells were treated as in D for the indicated time periods. The phosphorylation levels of p65 (Ser276) and CREB (Ser133) were evaluated by Western blot. (**F**) Cells were treated as in A for the detection of phospho-CREB (Ser133) and β-actin by Western blot. (**G**) THP-1 cells were transfected with 10 nM of scramble siRNA (NC) or CREB-targeting siRNA for 48 h. The whole cell lysates were obtained to detect CREB levels using Western blot. (**H**) Cells transfected with siRNA were stimulated as in A. After 24 h of LPS stimulation, culture supernatants were collected to measure levels of secreted IL-8 by ELISA. *p < 0.05 (n = 3).

To determine phosphorylation levels at Ser276 and Ser536 of the NF-κB p65 subunit, THP-1 cells were pre-treated with anti-BAFF mAb, and then the cells were stimulated with LPS. LPS-induced phosphorylation of Ser276 was increased by pre-treatment with anti-BAFF mAb when compared to cells pre-treated with isotype matching mouse IgG (mIgG). In contrast, phosphorylation at Ser536 was not affected (Fig. 5A). Since phosphorylation of Ser276 is required for its interaction with CBP, a co-immunoprecipitation analysis was performed. Interaction between phospho-p65 and CBP was enhanced by simultaneous stimulation of TLR4 and BAFF (Fig. 5B).

Acetylation of NF-κB is required for its activity as a transcription factor, and this is mediated by the acetyltransferase activity of CBP, which acetylates Lys310 of the p65 subunit^29^. Analysis of NF-κB acetylation indicated that LPS-induced acetylation of p65 was enhanced by anti-BAFF mAb pre-treatment (Fig. 5C). These data indicate that synergistic activation of NF-κB is possible through enhanced interaction of NF-κB with CBP, which then acetylates NF-κB.

Since mitogen- and stress-activated protein kinase (MSK)1 is the down-stream target of MAPK and is responsible for phosphorylation of p65 Ser276^31^, MSK1 involvement was investigated next. As shown in Fig. 5D, the MSK-specific inhibitor SB747651A blocked the synergistic expression of IL-8 in cells stimulated with LPS and anti-BAFF mAb. In addition, phosphorylation of Ser276 was blocked in the presence of SB747651A (Fig. 5E).

### Phosphorylated CREB interacts with the NF-κB/CBP complex and plays a positive role in the synergistic induction of proinflammatory mediators

Immunoprecipitation analysis indicated that crosstalk between TLR4- and BAFF-mediated signaling also increased the amount of phospho-cyclic AMP-response element binding protein (CREB) in the complex containing CBP and NF-κB (Fig. 5B). Thus, we tried to define the role of CREB in the crosstalk between TLR4- and BAFF-mediated signaling pathways. First, the level of CREB Ser133 phosphorylation was evaluated after stimulation of TLR4 and BAFF. Pre-treatment with anti-BAFF mAb alone induced CREB phosphorylation (Fig. 5F). LPS-induced CREB phosphorylation was also enhanced in cells pre-treated with BAFF-specific antibody. In order to determine whether CREB plays a positive or negative role in the crosstalk, CREB expression was suppressed by CREB-specific siRNA transfection (Fig. 5G). When stimulated with LPS and anti-BAFF mAb, the synergistic induction of IL-8 expression was significantly reduced in CREB siRNA-transfectants when compared to control transfectants (Fig. 5H). These results indicate that the formation of complexes containing phospho-NF-κB, phospho-CREB, and CBP was enhanced as a result of the crosstalk between signals initiated from TLR4 and BAFF, and that this complex plays a positive role in the synergistic induction of proinflammatory mediators.

Since previous reports indicated that the phosphorylation of CREB can be mediated by MSK in macrophages^32^, we determined the levels of CREB phosphorylation in the presence of SB747651A. As shown in Fig. 5E, inhibition of MSK activity results in a reduction in CREB phosphorylation, indicating that the MAPK/MSK signaling pathway can contribute to the phosphorylation of CREB.

### The PI3K/AKT/CREB pathway is enhanced by crosstalk between TLR4- and BAFF-mediated signaling pathways

**Figure 6:**
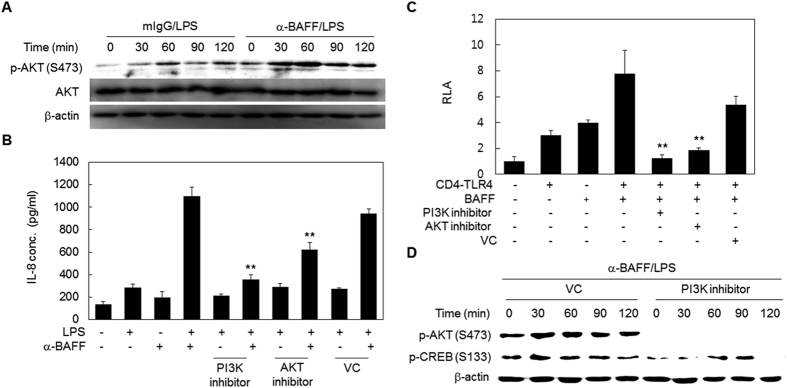
The PI3K/AKT/CREB pathway contributes to the synergistic activation of NF-κB and expression of IL-8. (**A**) THP-1 cells were pre-treated with anti-BAFF or mIgG (1 μg/ml) for 30 min and then stimulated with 100 ng/ml of LPS for the indicated times. Total cell lysates were tested for the levels of phospho-AKT(Ser473), AKT, and β-actin by Western blot. (**B**) THP-1 cells were pre-treated with 5 μM LY294002 (PI3K inhibitor), 10 μM MK2206 (AKT inhibitor) or 0.05% DMSO (VC) for 1 h. The cells were treated with anti-BAFF (1 μg/ml) for 30 min and then stimulated with LPS (100 ng/ml) for 24 h. The levels of secreted IL-8 in culture supernatants were determined by ELISA. **p < 0.01 when compared with corresponding positive control samples stimulated in the absence of inhibitors (n = 6). (**C**) HEK 293 T cells were co-transfected with an NF-κB luciferase construct, reference reporter construct, and BAFF and CD4-TLR4 expressing vectors. After 3 h, the cells were treated with 20 μM LY294002 (PI3K inhibitor), 10 μM MK2206 (AKT inhibitor) or 0.2% DMSO (VC). After 21 h, relative luciferase activities (RLA) were measured. **p < 0.01 when compared with cells co-transfected with BAFF and CD4-TLR4 expressing vectors (n = 6). (**D**) THP-1 cells were pre-treated with 20 μM LY294002 (PI3K inhibitor) or 0.2% DMSO (VC) for 1 h. The cells were stimulated with LPS (100 ng/ml) for indicated times in the presence of anti-BAFF mAb (1 μg/ml). The phosphorylation levels of AKT and CREB were analyzed by Western blot.

Activated phosphatidylinositol-3-kinase (PI3K) has been found to have a regulatory function in inflammation and many pro- and anti-inflammatory agents exert their effect through activation or inhibition of PI3K^33^,^34^,^35^. In order to determine the role of PI3K-AKT signaling in the synergism, THP-1 cells were sequentially treated with anti-BAFF mAb and LPS, and then activation of the PI3K-AKT signaling pathway was evaluated by Western blot detection of the phosphorylation levels of AKT, the main substrate of PI3K. LPS-induced phosphorylation of AKT was enhanced by pre-treatment with anti-BAFF mAb (Fig. 6A). In addition, synergistic expression of IL-8 by treatment of LPS and anti-BAFF mAb were diminished by pre-treatment with LY294002 or MK2206 (inhibitors for PI3K or AKT, respectively) (Fig. 6B).

The role of the PI3K/AKT signaling pathway in the crosstalk was then examined using an NF-κB-luciferase assay. Enhanced NF-κB activity in cells transfected with BAFF and CD4-TLR4 expression vector was significantly reduced in the presence of LY294002 or MK2206 (Fig. 6C). Since the PI3K/AKT pathway can lead to the phosphorylation of CREB^36^,^37^, CREB phosphorylation levels were then tested in the presence of LY294002. As shown in Fig. 6D, LY294002 treatment resulted in the suppression of CREB phosphorylation. These results indicate that the PI3K-AKT pathway is enhanced during the crosstalk, and that this contributes to the phosphorylation and subsequent activation of CREB.

## Discussion

**Figure 7:**
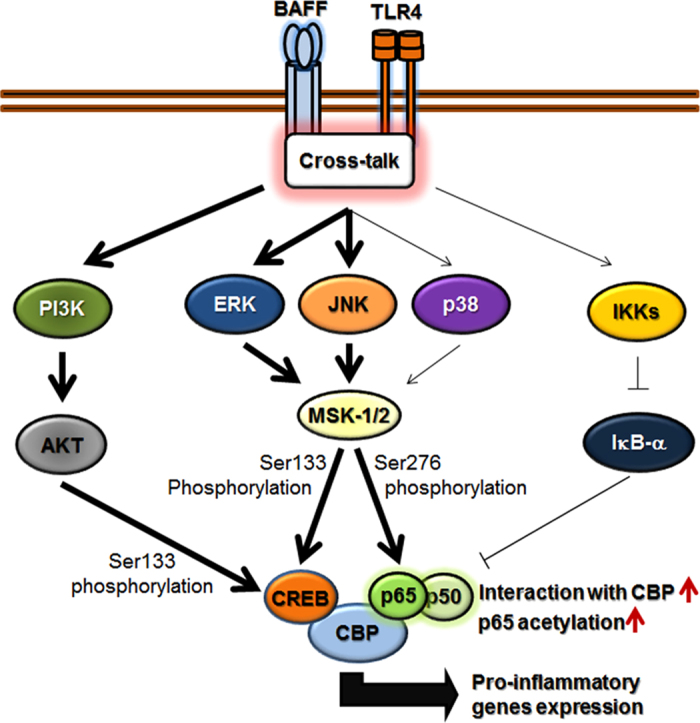
A diagram showing the signaling pathways responsible for the synergistic induction of proinflammatory mediators. Simultaneous stimulation of BAFF and TLR4 appears to enhance NF-κB activation through phosphorylation of the NF-κB 65 subunit (Ser276) through the MAPK(ERK/JNK)/MSK pathway, without affecting the LPS-induced degradation of IκB or NF-κB nuclear translocation. NF-κB then interacts with CBP for subsequent acetylation. CREB is phosphorylated through the MAPK/MSK and PI3K/AKT pathways, and the resulting phosphorylated CREB interacts with the NF-κB/CBP complex. The trimeric complex composed of NF-κB, CBP, and CREB then plays a positive role in the transcriptional activation of pro-inflammatory genes. (Signaling pathways enhanced by the crosstalk are indicated with bold lines).

The stimulation of BAFF enhanced LPS-induced expression of pro-inflammatory mediators such as MMP-9, IL-8, TNF-α, and MCP-1. Analyses of the activation status of signaling adaptors indicated that the enhancing effect of BAFF was possible through its activation of signaling adaptors that are also involved in LPS-induced signaling pathways (Fig. 7). These signaling adaptors include ERK and JNK MAPKs and PI3K, which subsequently activated MSK1 and AKT, respectively. MSK activation led to the enhancement of Ser276 phosphorylation in the NF-κB p65 subunit, which then interacts with CBP. Acetyltransferase activity of CBP then acetylates the p65 subunit which, as a result, has higher transcription activating activity. On the other hand, both the MAPK/MSK and PI3K/AKT pathways appear to enhance Ser133 phosphorylation of CREB, which then joins the CBP/NF-κB complex. Based on the fact that siRNA-mediated down-regulation of CREB resulted in the reduction of the enhancing effect of BAFF, the NF-κB/CREB/CBP trimeric complex appears to have a strong positive effect on pro-inflammatory gene activation (Fig. 7).

Previously, we demonstrated that treatment with anti-BAFF mAb at high concentration (10 μg/ml) induced the expression of MMP-9 and IL-8 through activation of ERK MAPK and NF-κB^10^. In this high-dose treatment of anti-BAFF mAb, NF-κB was activated through phosphorylation/degradation of IκB and the subsequent nuclear translocation of NF-κB. The BAFF-mediated activation of NF-κB, however, did not result in TNF-α or MCP-1 induction. In our current experiment, a low concentration of anti-BAFF mAb (1 μg/ml) was used. This concentration is not strong enough to induce high level expressions of these proinflammatory mediators. However, when this low concentration of anti-BAFF mAb was used in combination with LPS, a strong enhancing effect on the expression of MMP-9, TNF-α, IL-8, and MCP-1 was detected. When compared with the high-dose antibody treatment, the low-dose antibody treatment appears to activate different signaling adaptors, with the exception of ERK MAPK, which is activated in both cases. These data indicate that different signaling adaptors can be activated, depending on the intensity of BAFF stimulation.

The activation of NF-κB is tightly regulated at the multiple levels by various cellular mechanisms. The main control of NF-κB activity is the regulation of its location within the cell. When there is no activation signal, NF-κB is detained in the cytosol in complex with IκB. Inflammatory signaling induces the phosphorylation and subsequent degradation of IκB and the resulting free NF-κB translocates into the nucleus for the transcriptional activation of various genes involved in inflammation^38^,^39^. The BAFF-mediated signaling pathway does not appear to change the LPS-induced phosphorylation/degradation of IκB nor the nuclear translocation of NF-κB.

The transcription activating function of NF-κB is affected by chemical modifications including phosphorylation, methylation, and acetylation at specific amino acid residues on its p65 subunit (RelA)^40^,^41^. Phosphorylation in the p65 subunit is well established as a crucial modification for the activation of NF-κB. Phosphorylation at Ser536 increases the transcriptional activity of NF-κB^42^,^43^. Phosphorylation at Ser276 is required for interaction with the co-activator CBP, which controls gene expression through modifying chromatin activity through acetylation of various substances, including histone N-terminal tails and NF-κB^28^,^44^. BAFF-mediated signaling enhanced the phosphorylation at Ser276, but not at Ser536, and, as a result, enhanced the interaction between NF-κB and CBP.

Acetylation also plays an important role in regulating NF-κB activity^29^. Acetylation of the p65 subunit at Lys310, and to a lesser extent at Lys221, modulates NF-κB activity, such as transcriptional activation, DNA binding, and assembly with IκBα. Enhanced phosphorylation at Ser276 of the NF-κB p65 subunit and the subsequent increase in its interaction with CBP was observed in THP-1 cells after sequential treatment with anti-BAFF mAb and LPS. The simultaneous increase in p65 acetylation levels indicates that enhanced interaction between CBP and NF-κB leads to CBP-mediated acetylation of NF-κB. This is in agreement with previous reports showing the direct action of CBP in NF-κB acetylation^29^.

CREB is commonly considered as an anti-inflammatory transcription factor in innate immune responses, since activated-CREB induces the anti-inflammatory cytokine IL-10 and can suppress pro-inflammatory gene expression through competition with p65 for interaction with CBP^45^,^46^. However, some reports indicate that CREB is involved in pro-inflammatory gene expression^47^,^48^. Furthermore, it can cooperatively act with NF-κB to induce gene expression^49^,^50^. Our experimental evidence indicates that CREB phosphorylation occurs via both the MAPK/MSK and PI3K/AKT pathways. Co-immunoprecipitation analysis and siRNA-mediated suppression of CREB expression indicated that phospho-CREB has a positive effect on pro-inflammatory gene expression in the crosstalk between BAFF- and TLR4-mediated signaling by forming trimeric complexes containing NF-κB, CBP, and CREB.

The PI3K-AKT signaling pathway has been reported to be an essential regulator of inflammatory responses. However, the precise effect of PI3K-AKT signaling on inflammation is highly controversial. Some reports suggest an anti-inflammatory role for PI3K-AKT signaling through its control of signaling molecules such as MAPKs and GSK-3β^33^. In contrast, others claim that PI3K-AKT signaling promotes inflammatory immune responses by inducing NF-κB activation^34^,^35^. Our analyses indicate that the PI3K/AKT signaling leads to phosphorylation/activation of CREB, and that the PI3K/AKT/CREB pathway is positively involved in the enhancing effect of BAFF on LPS-induced responses. It is highly likely that the effect of PI3K/AKT activation on NF-κB activity is an indirect effect through CREB, which enhances NF-κB activity via formation of NF-κB/CREB/CBP complexes.

It is interesting that the crosstalk between signals initiated by members of the TNFSF and TLR4 has been previously reported in the case of 4-1BB ligand (4-1BBL)^21^. Kang *et al*., demonstrated that the reverse signaling generated from 4-1BBL sustained TNF production through CREB and C/EBP in macrophages. The involvement of NF-κB, however, was not detected in the crosstalk between 4-1BBL- and TLR4-mediated signaling pathways.

Our results demonstrate, for the first time, that the crosstalk between the signaling initiated from BAFF may affect LPS-induced inflammatory activation of macrophages. This crosstalk resulted in the synergistic activation of various pro-inflammatory mediators. This synergistic activation was possible through BAFF-mediated activation of signaling adaptors that are shared by LPS-induced signaling pathways. These data demonstrate the existence of a complex network of signaling pathways in inflammatory activation of macrophages that will be helpful in the future development of immunomodulatory molecules to regulate the inflammatory activation of macrophages and, possibly, other white blood cells.

## Materials and Methods

### Antibody and reagents

The mAb against human BAFF were purchased from Immunomics (clone T7-132) (Ulsan, KOREA), R&D Systems (clone 148725) (Minneapolis, MN, USA), and Santa Cruz (clone B418) (Santa Cruz, CA, USA). Mouse IgG2a was purchased from BD Biosciences (San Jose, CA, USA). SB203580 and LY294002 were acquired from Calbiochem International Inc. (La Jolla, CA, USA). U0126, rabbit polyclonal antibodies to ERK-1/2 (p42/44 MAPK), phospho-ERK-1/2 (Thr202/Tyr204), IκB-α, p38 MAPK, phospho-p38 MAPK (Thr180/Tyr182), JNK MAPK, phospho-AKT (Ser473), and AKT, rabbit mAbs to CREB, phospho-NF-κB p65 (Ser536) (93H1), and acetyl-NF-κB p65 (Lys310) and mouse mAbs to phospho-IκB-α (Ser32/36) (5A5), phospho-JNK MAPK (Thr183/Tyr185) (G9), and phospho-CREB (Ser133) (1B6) were purchased from Cell signaling (Danvers, MA, USA). Mouse mAb to p65, rabbit polyclonal antibodies to phospho-p65 (Ser276) and CBP, goat polyclonal antibody to β-actin, and siRNAs specific for BAFF and CREB were obtained from Santa Cruz. Bacterial LPS and SP600125 was purchased from Sigma (St. Louis, MO, USA). MK2206 were obtained from Selleck Chemicals (Houston, TX, USA). SB747651A was purchased from R&D Systems (Minneapolis, MN, USA). DharmaFECT 1 siRNA transfection reagent was obtained from Dhamacon, Inc (Lafayette, CO, USA).

### Cell culture and flow cytometric analysis

The human monocytic leukemia cell line, THP-1, was grown in RPMI 1640 (WelGENE Inc., Daegu, Korea), supplemented with 10% fetal bovine serum (FBS), 0.05 mM β-mercaptoethanol, glucose, and streptomycin-penicillin at 37 °C in the presence of 5% CO_2_. For activation, cells were seeded (1.0 × 10^6^ cells in 1 ml of RPMI medium) in 24-well plates and pre-treated with 1 μg/ml of anti-BAFF or mIgG for 30 min in the presence or absence of appropriate inhibitors. Cells were then stimulated with 0.01–1 μg/ml of LPS for 0.5–24 h. The human embryonic kidney cell line 293 T (HEK 293 T) was grown in DMEM (WelGENE Inc.,), supplemented with 10% FBS and streptomycin-penicillin at 37 °C in the presence of 5% CO_2_. Flow cytometric analysis was performed using the FACSVerse system (Becton-Dickinson, Mountain View, CA, USA). To investigate the expression levels of BAFF in THP-1 cells, the cells (5.0 × 10^5^) were washed twice with cold phosphate buffered saline (PBS) and then stained with 1 μg/ml of anti-BAFF mAb or 1 μg/ml mIgG for 30 min on ice. The cells were then washed twice with cold PBS and incubated with 10 μg/ml of FITC-conjugated goat anti-mouse secondary antibody for 15 min on ice. After incubation, the cells were washed twice with cold PBS and re-suspended with cold PBS. The mIgG staining sample was used for background fluorescence. The fluorescence profiles of 1.0 × 10^4^ cells were collected for analysis.

### Gelatin zymography and enzyme-linked immunosorbent assay (ELISA)

The activity of MMP-9 in the culture supernatant was determined by substrate gel electrophoresis. Zymography using sodium dodecyl sulfate-polyacrylamide gel electrophoresis (SDS-PAGE) in gel containing 0.1% gelatin was performed as previously described^51^. Briefly, culture supernatants were collected after 24 h, mixed with FOZ buffer (4% SDS, 20% glycerol, 0.01% bromophenol, 0.125 M Tris-Cl), and electrophoresed on 10% polyacrylamide gels. The gels were then sequentially treated with 2.5% Triton X-100 for 40 min to remove SDS, distilled water for 40 min to remove Triton X-100, and digestion buffer (50 mM Tris-Cl, pH 7.6, 0.15 M NaCl, 10 mM CaCl_2_, 0.02% NaN_3_) for 12 h at 37 °C. The gels were finally stained with 0.1% Coomassie Brilliant Blue R-250 (USB Corporation, Cleveland, USA) and destained to visualize the protein bands. The cytokine concentrations in culture supernatants were measured using sandwich ELISA kits (eBioscience, San Diego, CA, USA). ELISA was performed according to the manufacturer’s protocol. Colorimetric changes were detected using a microplate reader set to 450 nm (corrected by the absorption at 540 nm). Measurements were performed in triplicate, and the detection limit was < 10 pg/ml.

### Reverse transcription polymerase chain reaction (RT-PCR)

Cells were collected and total cellular RNA was extracted. Isolated RNAs were treated with RNase free DNase I (Takara Bio Inc., Otsu, Shiga, Japan) and then used for cDNA synthesis, which was conducted using Diastar^TM^ RT kit (SolGent, Daejeon, Korea) according to the manufacturer’s protocol. For PCR, primers were prepared for BAFF (370 bp; forward primer, GGT CCA GAA GAA ACA GTC AC; reverse primer, GGA GTT CAT CTC CTT CTT CC), GAPDH (391 bp; forward primer, ATC ACT GCC ACC CAG AAG AC; reverse primer, TGA GCT TGA CAA AGT GGT CG). The expression levels of GAPDH were used as an internal control. After heating at 94 °C for 5 min, PCR was performed under the following conditions: 35 cycles of 94 °C for 1 min, 57 °C for 1 min, and then 72 °C for 1 min, followed by a final extension at 72 °C for 7 min. PCR products were then resolved on a 1.2% agarose gel stained with ethidium bromide. Quantitative RT-PCR was performed and analyzed by StepOnePlus (Applied Biosystems, Foster City, CA, USA) with SYBR Premix Ex Taq (Takara Bio Inc., Otsu, Shiga, Japan) and specific primers for TNF-α (forward primer, GGA GAA GGG TGA CCG ACT CA; reverse primer, CTG CCC AGA CTC GGC AA), IL-8 (forward primer, ATA AAG ACA TAC TCC AAA CCT TTC CAC; reverse primer, AAG CTT TAC AAT AAT TTC TGT GTT GGC), MCP-1 (forward primer, ACT CTC GCC TCC AGC ATG AA; reverse primer, TTG ATT GCA TCT GGC TGA GC), MMP-9 (forward primer, TTC TAC GGC CAC TAC TGT GCC T; reverse primer, AAT CGC CAG TAC TTC CCA TCC T) and GAPDH (forward primer, TGG GCT ACA CTG AGC; reverse primer, GGG TGT CGC TGT TGA AGT CA). The threshold cycle (Ct) values obtained for each reaction were normalized using the GAPDH Ct values.

### Luciferase assay

HEK 293 T cells were seeded (1.0 × 10^4^ cells in 100 μl of DMEM medium, triplicate/sample) in 96-well plates and incubated overnight before transfection. A mixture containing 400 ng/well of total DNA and 2.5 μl of Superfect transfecting reagent (Qiagen, Valencia, CA) was suspended in 100 μl of antibiotics-free DMEM medium was added into the culture wells. After 24 h, cell lysates were obtained in passive lysis buffer (Promega, Madison, WI), and luciferase activities were measured using the Dual-Luciferase reporter assay system (Promega). Relative luciferase activity (RLA) was determined by normalization with Renilla luciferase activity. Expression constructs for CD4-TLR4, the death domain of MyD88, wild type TRIF, the luciferase reporter gene under the control of NF-κB binding sites, and the Renilla-luciferase construct for a transfection control were described previously^52^

### Immunocytochemistry

To detect nuclear translocation of p65, THP-1 cells were washed twice with cold PBS and attached on cover slips that were coated with poly-L-lysine (Sigma). Attached cells were fixed with 4% paraformaldehyde for 15 min at room temperature (RT) and permeabilized with 0.5% Triton-X in PBS for 15 min. The cells on cover slip were incubated with anti-NF-κB p65 mAb (4 μg/ml) at 4 °C overnight and then incubated with 10 μg/ml of FITC-conjugated goat anti-mouse secondary antibodies at RT for 1 h. Cover slips were mounted with DAPI-containing mounting solution for observation with a fluorescence microscope and enumeration.

### Western blot

THP-1 cells were seeded (1.0 × 10^6^ cells in 1 ml of RPMI 1640 medium) in 24-well plates and pre-treated with antibodies. After 30 min, cells were stimulated with LPS for indicated times and then cells were collected by centrifugation (8,000 rpm for 3 min at 4 °C). The cells were lysed in 100 μl of NP-40 (IGEPAL CA-630) lysis buffer (150 mM NaCl, 1% IGEPAL CA-630, 50 mM Tris, pH 8.0) containing protease inhibitor cocktail (Calbiochem, San Diego, CA, USA), sodium orthovanadate (Na_3_VO_4_) (Sigma), and deacetylation inhibition cocktail (Santa Cruz). The debris were then removed from total cell lysates by centrifugation (12,000 rpm for 15 min at 4 °C), and a Western blot was performed as described previously^10^. The specific bands on membranes were visualized by exposure to a Davinch-chemi™ Chemiluminescence Imaging System (CoreBio, Seoul, Korea).

### Immunoprecipitation

THP-1 cells were seeded and pre-treated as in Western blot analysis. After 30 min, cells were stimulated with LPS for 1 h and then cells were collected by centrifugation (8,000 rpm for 3 min at 4 °C). The cells were lysed with 150 μl of non-denaturing lysis buffer (20 mM Tris HCl pH 8.0, 137 mM NaCl, 10% glycerol, 1% NP-40, and 2 mM EDTA in H_2_O) containing protease inhibitor cocktail (Calbiochem) and Na_3_VO_4_ (Sigma). Anti-CBP or anti-p65 antibody was added to each of the cell lysates (150 μl) and the protein-antibody mixtures were then incubated at 4 °C overnight. The protein-antibody mixtures were incubated with 50 μl of protein A/G-conjugated magnetic beads (GenScript, NJ, USA) in non-denaturing lysis buffer on a rocker at 4 °C overnight. The protein-antibody-bead complexes were separated from whole cell lysates using a magnetic rack and then were washed twice with cold PBS. Finally, elution buffer (1% SDS, 50 mM Tris HCl, 100 mM DTT in H_2_O, pH 7.4) was added to the precipitated complex, and mixtures were boiled at 95 °C for 5 min to separate protein from beads. Then, supernatants were collected to use in a Western blot.

### Transfection of siRNA

THP-1 cells (2.0 × 10^5^ cells) were pre-seeded in 6-well plates with antibiotic-free culture medium. After 16 h, the culture medium was replaced with flesh antibiotic-free medium. Then, the cells were transfected with siRNA at a concentration of 100 nM using DharmaFECT 1 siRNA transfection reagent according to the manufacturer’s protocol. Transfected cells were collected and used for analysis of mRNA and protein levels at 24 or 48 h after transfection, respectively.

### Statistical analysis

All data are presented as the mean values ± SD, with the number of independent experiments indicated in the figure legends. Statistical difference of two means between groups in ELISA data was determined by two-tailed asymptotic general independence test. Asymptotic general independence test was performed using Coin package in R statistical language. Luciferase assay results were statistically evaluated using two-tailed Student’s *t*-test. Normal distribution of data sets was assumed by Kolmogorov-Smirnov (K-S) test. Student’s *t*-test and K-S test were performed using GraphPad Prism version 5.01. Statistical power was determined by post-hoc analysis in G*Power 3.1. Differences were considered significant at *p* < 0.05 and power ≥ 0.8 (Student’s *t*-test) or 0.95 (asymptotic general independence test).

## Additional Information

**How to cite this article**: Lim, S.-G. *et al*. Crosstalk between signals initiated from TLR4 and cell surface BAFF results in synergistic induction of proinflammatory mediators in THP-1 cells. *Sci. Rep.* **7**, 45826; doi: 10.1038/srep45826 (2017).

**Publisher's note:** Springer Nature remains neutral with regard to jurisdictional claims in published maps and institutional affiliations.
